# Network-based biomarkers enhance classical approaches to prognostic gene expression signatures

**DOI:** 10.1186/1752-0509-8-S4-S5

**Published:** 2014-12-08

**Authors:** Rebecca L Barter, Sarah-Jane Schramm, Graham J Mann, Yee Hwa Yang

**Affiliations:** 1 School of Mathematics and Statistics at The University of Sydney, F07, The University of Sydney, NSW, 2006, Australia; 2 Westmead Millennium Institute at The University of Sydney, 176 Hawkesbury Road, Westmead, NSW, 2145, Australia; 3 Melanoma Institute Australia, 40 Rocklands Rd, North Sydney, NSW, 2060, Australia

**Keywords:** biomarker, gene expression, gene-set, melanoma, microarray, network, ovarian cancer, prognosis, protein-protein interaction

## Abstract

**Background:**

Classical approaches to predicting patient clinical outcome via gene expression information are primarily based on differential expression of unrelated genes (single-gene approaches) or genes related by, for example, biologic pathway or function (gene-sets). Recently, network-based approaches utilising interaction information between genes have emerged. An open problem is whether such approaches add value to the more traditional methods of signature modelling. We explored this question via comparison of the most widely employed single-gene, gene-set, and network-based methods, using gene expression microarray data from two different cancers: melanoma and ovarian. We considered two kinds of network approaches. The first of these identifies informative genes using gene expression and network connectivity information combined, the latter drawn from prior knowledge of protein-protein interactions. The second approach focuses on identification of informative sub-networks (small networks of interacting proteins, again from prior knowledge networks). For all methods we performed 100 rounds of 5-fold cross-validation under 3 different classifiers. For network-based approaches, we considered two different protein-protein interaction networks. We quantified resulting patterns of misclassification and discussed the relative value of each relative to ongoing development of prognostic biomarkers.

**Results:**

We found that single-gene, gene-set and network methods yielded similar error rates in melanoma and ovarian cancer data. Crucially, however, our novel and detailed patient-level analyses revealed that the different methods were correctly classifying alternate subsets of patients in each cohort. We also found that the network-based *NetRank *feature selection method was the most stable.

**Conclusions:**

Next-generation methods of gene expression signature modelling harness data from external networks and are foreshadowed as a standard mode of analysis. But what do they add to traditional approaches? Our findings indicate there is value in the way in which different subspaces of the patient sample are captured differently among the various methods, highlighting the possibility of 'combination' classifiers capable of identifying which patients will be more accurately classified by one particular method over another. We have seen this clearly for the first time because of our in-depth analysis at the level of individual patients.

## Background

Gene expression signatures have long been heralded for the way in which they might revolutionise clinical practice in terms of personalising medicine: a regime in which clinicians have the ability to segment heterogeneous subsets of patients according to the treatment options from which they are expected to derive the most benefit [1]. However, despite some 15 years of rigorous investigation across a multitude of cancer types there is a worrying dearth in the translation of this particular class of biomarker [2-5]. This situation presents a clear and pressing opportunity for the critical examination and ongoing development of methods to: 1) select clinically relevant features from gene expression microarray information; and, 2) use a quantitative measure of those features to define a model which can be used to accurately distinguish between groups of interest *e.g*., longer versus shorter survival.

Identification of a prognostic gene expression signature can be considered, in essence, a two-step procedure. First, the informative features are identified, for example by ranking all potential features in such a way that assigns the top ranks to those that differ most between the groups of interest. The top-ranked features are then selected for classification, which is the second step in the signature building process. The classifier itself is an algorithm - or a function of several variables or features - that can be mapped to a categorical space, such as the binary space consisting of longer (good prognosis, GP) or shorter (poor prognosis, PP) survival after diagnosis with cancer, as we explore herein.

**Figure 1:**
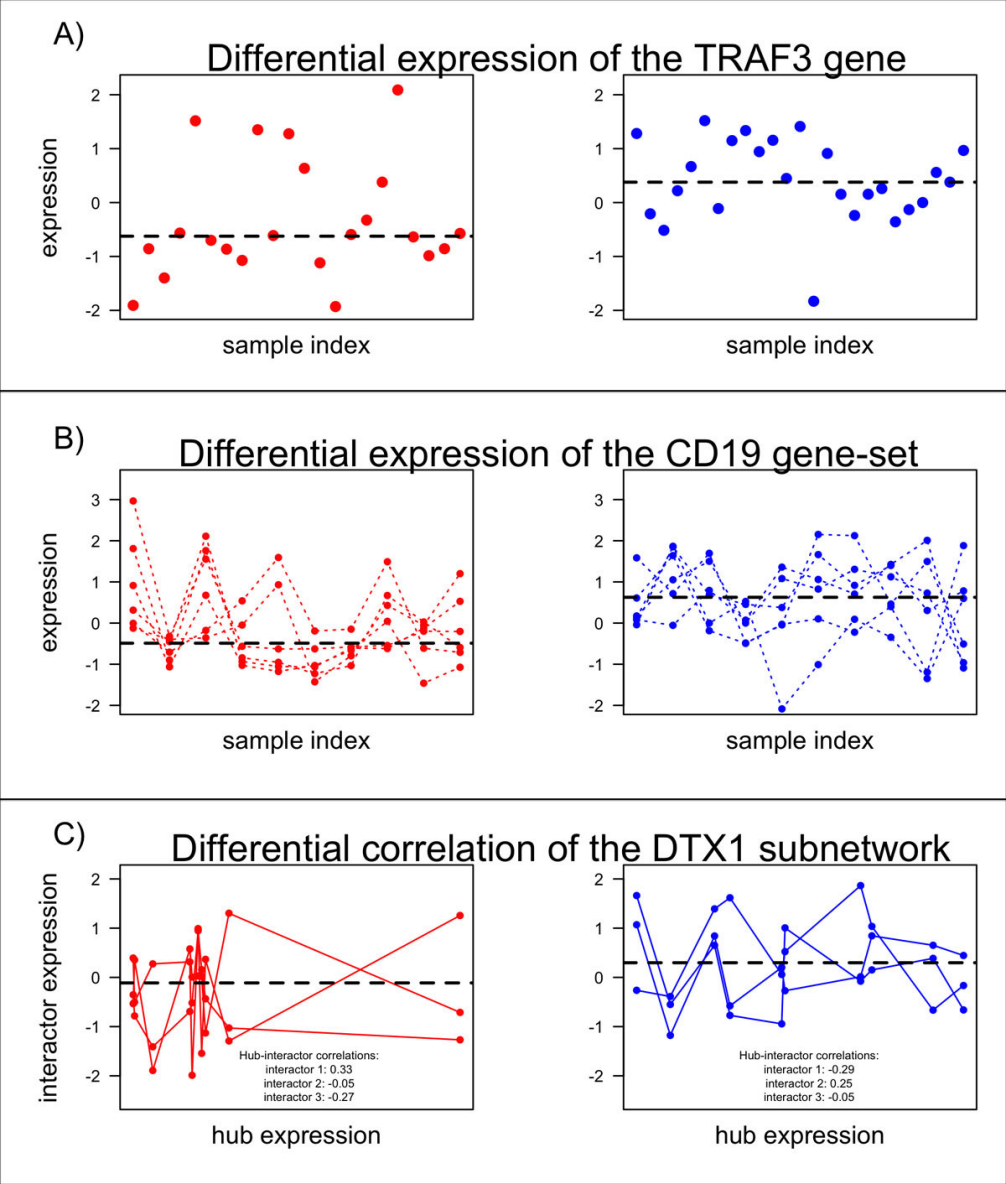
**Examples of informative features which differ between the PP class (red) and GP class (blue)**. These examples were obtained using the melanoma data set and the iRefWeb network. A) presents the differential expression of the TRAF3 gene (the x-axis corresponds to the samples and the y-axis corresponds to the expression values), B) presents the differential (median) expression of the CD19 gene-set which consists of 6 genes (the x-axis corresponds to the samples and the y-axis corresponds to the expression values), and C) presents the differential correlation of the DTX1 hub sub-network (for visual simplicity, we present the hub gene expression (x-axis) versus interactor gene expression (y-axis) for three of the five edges in the DTX1 hub sub-network and for 10 samples from each class). The hub-interactor correlations for each hub-interactor pair are presented. Image adapted from [6].

The most widely used methods in gene expression signature modelling to date can be partitioned into two broad groups [6]. The first of these approaches is the 'single-gene' method in which no prior or external information is incorporated into the analysis in any way. The features for these methods are individual genes identified, for example, via differential expression analysis (Figure 1A). In the classification step of the single-gene approach, a classifier is built which takes the expression values of these informative genes as the input, and outputs the predicted class of the sample. The second general approach, termed the 'gene-set' method, involves grouping genes together into sets to be used as classification features. Such genes are typically related via co-membership of a biochemical pathway or other biologic feature. The classification features for gene-set methods are usually the sets of genes themselves that are considered to differ between the groups of interest in some way (Figure 1B). In this case a measure that can quantify a gene-set independently for each sample, analogous to the way in which single genes are quantified by their expression values, is needed. For clarity we note that gene-set approaches are often referred to as pathway methods and sometimes, confusingly, as network methods. However the distinction between gene-set methods and network methods in this study is that gene-set methods do not incorporate any network edge information (*vide infra*) into the feature selection procedure or the feature values.

In recent years, network-based methods of gene expression analysis have grown in popularity for their capacity to capture or explain emergent properties such as biological heterogeneity, modularity, or phenotypic variability [7]. A network, or a graph as it is known in a mathematical context, consists of a set of nodes, *V*, and a set of edges, *E*, between the nodes. A network can be described by an adjacency matrix **W **= (*w_ij_*), in which a non-diagonal entry, *w_ij _*is the number of edges from node *i *to node *j *(equal to zero if no such edge exists). The degree of a node is defined to be the number of edges incident to the node and a hub is a highly connected node which we defined as node with at least 5 interactors in the network, consistent with the definition of a hub in prior related works [8-10]. A sub-network is a network whose vertex set is a subset of *V *and whose edge set is a subset of *E *restricted to this vertex set *i.e*., a smaller portion of the entire known network. A hub sub-network is a sub-network consisting of a hub node and its immediate interactors together with the hub-interactor edges. The concept of a network is easily translated into a biological setting with genes represented by nodes and relationships between genes (such as interactions) represented by edges (Figure 1C). In this study we examine protein-protein interaction (PPI) networks in which nodes correspond to protein-coding genes (representing the mRNA information) and the existence of an edge between two such genes implies that the proteins coded for by those genes interact in a direct and physical manner with each other in a biological setting.

A number of approaches exist involving the utilisation of PPI networks in gene expression signature modelling [7]. We examine two increasingly popular strategies herein [8]. The first approach [11] focuses on the network properties of individual genes. More specifically, individual genes are used as the classification features while the PPI network information is used in feature selection to identify the most informative genes. This is in contrast to the (non-network) single-gene method introduced above in which the classification features are individual genes, but with no utilisation of network information. The second network-based method focusing on informative sub-networks (rather than individual genes) is a particularly active area of investigation [8,10,12]. This approach involves mathematical integration of PPIs with gene expression information to identify differential network behaviour such as changes in correlation between the gene expression profiles of interacting gene pairs (Figure 1C). In this approach, the feature selection process involves identifying informative sub-networks such as those whose correlation structure differs between the groups of interest. The sub-networks examined in this study are of the hub-type described above. The classification features for this type of network approach are then defined as the informative hub sub-networks themselves or are extracted from them *e.g*., by considering the edges or the hub genes within the hub sub-network.

In this study we evaluated and compared the three fundamental kinds (single-gene, gene-set, and network-based) of gene expression biomarker modelling described above (also summarised below and in Table 1). Specifically, we explored their capacity to classify tumors according to phenotypes of distinct pathophysiological states and associated clinical outcomes in previously well-characterised melanoma [13,14] and ovarian cancer [15] cohorts. For statistical rigour, we employed three different classifiers each under 5-fold cross-validation scenarios: random forest (RF) [16], diagonal linear discriminant analysis (DLDA) [17] and support vector machines (SVMs) [18].

**Table 1:** Summary of feature selection methods analysed.

Approach	Method name	Feature selection	Classification Feature	Feature value	Existing method?	**Ref**.
**Single-gene**	*Mod-t*	Rank genes by p-value	Single gene	Expression value	Yes	Smyth *et al*. [30]
**Gene-set**	*Median expression*	Rank gene-sets by p-value	Gene-set	Median expression value of all genes in the gene-set	No	
**Network**	*NetRank*	Rank genes using *NetRank *algorithm	Single gene	Expression value	Yes	Winter *et al*.[11]
	*Taylor*	Rank sub-networks using Taylor's correlation-based measure	Sub-network edge	Expression difference between hub and interactor gene connected by the edge	Yes	Taylor *et al*. [8]
	*BSS/WSS*	Rank sub-networks using the *BSS/WSS *correlation-based measure	Single (hub) gene	Expression value of the gene	No	

Description of the methods assessed in terms of the way in which external information is incorporated, the feature selection procedure, the features used for classification, and a reference if the method is obtained from a previous publication.

Overall we aimed to critically evaluate the relative contribution of PPI network-based approaches (*cf*. the now classical single-gene and gene-set methods) to gene expression signature profiling in cancer prognostication. What was novel in our analytical approach, beyond evaluation of overall classification error rates and feature selection stability, was the modelling of findings both at the class-specific level and at the level of individual patients. The former process involved observing the average error rates over the poor prognosis samples and the good prognosis samples, while the latter analysis considered the proportion of cross-validation folds in which each patient was correctly classified. Via this unique approach, we made a number of observations. First, consistent with the findings of [19-21], the overall classification error rates across the single-gene, gene-set and network methods were comparable, suggesting that more complex models when assessed purely by classification error rates aren't necessarily more accurate. Second, we found that one particular network-based approach - *NetRank *[11] - was considerably more stable with respect to all other methods evaluated herein. This finding is in contrast with prior literature [19,20] in which network-based methods did not show an increase in stability *cf*. single-gene methods. It implies that incorporation of network information may lead to the identification of more stable gene expression signatures. Finally, by considering our results at the level of individual patients, we observed that different methods were capturing different subsets of the sample space *i.e*., the different approaches were correctly classifying different samples. This finding has the implication that there may in fact be a sub-class of patients who are more accurately classified by single-gene methods *cf*. network-based or gene-set methods and *vice-versa*. As a result, a possible future direction of research in this area could be the development of a combination classifier capable of identifying which patients will be more accurately classified by one particular type of method over the other.

For clarity we begin with a précis and motivation for the selection of each of the approaches evaluated in this study.

### Single-gene methods - a historical overview

The first methods to arise dealing with gene expression signatures were single-gene methods based on identification of individual differentially expressed (DE) genes. One initial and extremely simple approach to DE analysis was to calculate the gene expression fold-change [22-24], and define DE genes as those genes for which expression values exhibited the largest fold-change between two groups of interest. The next approach to DE analysis was to employ the *t*-test to identify genes having differential expression between groups of interest [25]. A standard *t*-test, however, did not adjust for individual gene variability. To counter this issue, modifications of the standard *t*-test were introduced [26]. Other statistical tests continued to arise for use on microarray data, including but not limited to ANOVA [27] and RVM [28]. However, the two most prominent single-gene methods to arise have been: 1) the significance analysis of microarrays (SAM) [29], which performs a non-parametric permutation-based test that does not assume equal variance of the genes; and, 2) the moderated *t*-statistic method (implemented by the limma package in R [30]), for which a robust linear model is fit to the expression profile for each gene. The moderated *t*-tests performed based on these models have increased degrees of freedom and the standard errors are moderated across the genes using a Bayesian model. The moderated *t*-statistic method is often considered to be the preferred method of differential gene expression analysis [31] and is thus the main single-gene method evaluated herein (Table 1 Figure 1A).

### Gene-set methods - the increasing sophistication of signature modelling

In the years following the rapid growth of the single-gene methods, approaches that considered sets of genes rather than individual genes began to emerge. Below we describe a few of the commonly used differential gene-set (or pathway) methods. The first of these approaches was gene set enrichment analysis (GSEA) , [32] for which an enrichment score is calculated for each gene-set reflecting the extent to which the gene-set is overrepresented at the extremes of the ranked list of DE genes. This method is based on the Wilcoxon rank sum test and there are a large number of variations including non-parametric methods such as gene set enrichment analysis rotation (GSEArot) [33], gene set analysis (GSA) [34]. Parametric methods include the random set method [35] and generally applicable gene set enrichment (GAGE) [36]. These gene-set methods are all examples of 'competitive gene-set methods' that utilize data not only from the gene-set of interest, but also data from outside the gene-set of interest. Another approach employs a modified multivariate Hotelling's *T*^2 ^test method [37] analogous to the single-gene univariate *t*-test approach. More recently, [38] described an algorithm, *Pathifier*, which uses expression data to calculate pathway deregulation scores.

It is important to note that the above methods offer an insight into the huge number of existing ways to identify informative or significant gene-sets but offer no means of translating these significant features into a classification setting. In particular, to use gene-sets as classification features, we must have a measure which can be used to quantify them independently for each sample in a manner analogous to the way in which individual genes are quantified by their expression values. The most obvious gene-set measure is to take the median expression of the genes within the set, which we will use herein. In the interests of using a feature selection procedure for which the median-expression measure will be able to capture the difference between the groups of interest, we utilised a gene-set analog of the single-gene moderated *t*-statistic method (Table 1 Figure 1B).

### Network approaches - a new class of tests

Although there are several methods that claim to be network methods in the literature [39,40], closer examination of several such methods reveal that they in fact fall into the category of gene-set methods since no network edge-information is utilized. Specifically, the majority of network-based methods focus on estimating network information from gene-expression data using probabilistic analyses, such as clustering, to infer co-regulated genes from co-expressed genes [41]. Recently, the weighted gene co-expression network analysis (WGCNA) approach [42] incorporated information corresponding to patient survival with their data-derived network. In this study we take a different approach by examining predefined networks, where we focus instead on incorporation of external PPI information. As stated above, we concentrate on two kinds of network approach; the first approach (which focuses on the network properties of individual genes) is one wherein individual genes are used as the classification features while PPI network information is incorporated into the feature selection procedure (Table 1). The primary network-based method of this type considered in this study is the *NetRank *algorithm [11]. *NetRank *is based on Google's *PageRank *algorithm which works by estimating the importance of a website by counting the number of links to it. However, *NetRank *is not the first method to attempt to replicate the *PageRank *algorithm in a genomic setting; [43] described an algorithm called *GeneRank *based on a similar idea. These algorithms estimate the relevance of a gene to the phenotype or 'class of interest' through consideration of both network connectivity and the gene's expression profile. There are several other examples of network methods of this type including the method described by [44] based on random walks, which utilises prior information on the relative importance of each gene. Another example is the CIPHER algorithm [45] which identifies disease genes by considering phenotypically similar diseases and a complete list of known disease gene-phenotype associations. A third example is the work of [46], aiming to measure the importance of genes by maximizing a likelihood function and identifying those genes that are the most highly connected. We note, however, that the above examples either focus on a different network-type [46] or require extra external information [44,45] and are therefore not evaluated in this study. In an example of the second type of network approach (which focuses on the properties of sub-networks, rather than individual genes), [8] identified sub-networks having a correlation structure that differed between conditions. The authors also offered a means of translating this feature selection method into a classification framework by using the edges from the differentially correlated networks as the classification features (Table 1 Figure 1C). We further adapted this idea of identifying differentially correlated networks by considering those with a large between-to-within sum of squares (BSS/WSS) ratio for the correlation values over the groups of interest. Other network approaches that focus on the identification of differential sub-networks include the method described by [47] which is based on the spectral decomposition of the gene expression profiles with respect to the eigen functions of the network. Similarly, [48] described an entropy-based method focusing on measuring the effect of randomness of single-genes while [49] proposed a different entropy-based method focusing on analysis of a heat kernel stochastic matrix. Unfortunately, the sub-network measures employed in these approaches are somewhat uninformative when applied to the structurally simple hub sub-networks as opposed to more complex sub-network structures. Therefore, in this study we look only at the network approach of [8] as well as the adapted BSS/WSS measure.

## Methods

### Gene expression microarray data sets

The melanoma microarray data set from [13] contains expression data for 17,552 genes for each of 47 patients with metastatic (stage III) melanoma, following filtering and processing as described in [13]. We previously [13] analysed the distribution of survival times to identify patients with more favourable (GP) and less favourable (PP) prognosis. These groups were defined as having time from surgery to death from melanoma greater than 4 years with no sign of relapse or less than one year. The data set contains 25 good prognosis samples and 22 poor prognosis samples (n_good_prognosis_melanoma_=25:n_poor_prognosis_melanoma_=22).

In addition to the melanoma data set we used the ovarian cancer data previously reported in [15] that describes a series of patients with stage III, high-grade primary papillary serous tumors of the ovary. We defined the poor prognosis class as patients who died within 2 years after surgery and the good prognosis class as patients alive more than 3 years after surgery. Following processing and filtering (per [15]), the data set consisted of expression data for 12,981 genes for each of 72 samples (n_good_prognosis_ovarian_=33:n_poor_prognosis_ovarian_=39).

### Protein-protein interaction (PPI) networks

We examined two previously analysed [10] PPI networks obtained from the MetaCore™ (from GeneGO™ Inc., version 6.6, build 28323) and iRefWeb (V3.4, March 2, 2011) [50] databases respectively. The MetaCore™ network contains 5,009 genes (4,069 of which appear in the melanoma microarray data set) and has 16,202 edges. The iRefWeb network comprises 7,256 genes (5,981 of which appear in the melanoma microarray data set and 5,623 of which appear in the ovarian cancer data set) and has 21,049 edges. Consistent with prior related literature [8], we defined a hub gene to be a gene with degree greater than or equal to 5 and a hub sub-network as the sub-network generated by considering a hub gene and its immediate interactors.

### Statistical analyses of single-gene, gene-set, and network-based methods

**Comparison study - overall strategy: **We evaluated and compared various single-gene, gene-set and network-based methods (Table 1). For each method, we first performed a feature selection step to identify informative explanatory variables or 'features'. These features were then used to build a model or 'classifier'. For all methods, the values used to quantify the classification features were calculated independently for each sample. Specifically, although values drawing information from more than one sample, such as by calculating correlation or *p*-values, can be used to perform feature selection, they cannot be used to define feature values for classification. We compare three different classifiers: 1) a tree-based RF classifier implemented using the randomForest package [16]; 2) an SVM classifier implemented using the e1071 package [51]; and, 3) a DLDA classifier implemented using the supclust package [52] in R [53]. For statistical rigour, all classification error rates were estimated using 100 rounds of 5-fold CV [53]. The mathematical details of the single-gene, gene-set and network methods performed in this study are given in Additional File 1 (Supplementary methods). However, for clarity we offer a brief description of each below.

**Comparison study - single-gene approach: **The single-gene method used was the moderated *t*-statistic, implemented via the lmFit and eBayes functions from the limma package [30] in R [53]. Briefly, we performed feature selection using a robust linear model that was fitted to the expression profile for each gene. To identify the most differentially expressed genes, moderated *t*-tests were performed based on these models. The moderated *t*-test had increased degrees of freedom and the standard errors were moderated across the genes using a Bayesian model [30] (Additional File 1 - Supplementary methods: *Single-gene approaches - moderated t-statistic*).

**Comparison study - gene-set approach: **The gene-set method analysed was the median expression approach in which gene-sets were quantified by the median expression of the genes contained within the set (inspired by the average expression measure described by [39] and work by [30]). The moderated *t*-statistic method was then applied to the gene-sets rather than the individual genes. That is, a robust linear model was fitted to each gene-set and moderated *t*-tests were performed to identify the most DE gene-sets. The gene-sets considered to be the most DE were then used as the classification features with the feature value defined to be their median expression value. In this study we used the PPI network information described above to define the gene-sets. In particular, gene-sets were defined as the sets of genes appearing together in a hub sub-network *i.e*., each gene-set contained a hub gene (a gene with at least 5 incident edges) and its immediate interactors (PPI partners). The network edges were ignored (Additional File 1 - Supplementary methods: *Gene-set approaches - median expression*).

**Comparison study - network-based approaches: **We undertook three network methods. The first of these, *NetRank *[11], is based on individual genes with the network information incorporated in the feature selection procedure. The *NetRank *algorithm iteratively assigned a rank to each gene, which depended both on the rank of all genes connected to it via an edge in the network and on the correlation of the gene's expression profile with survival time. The extent to which the network influenced the rank of each gene was determined by the value of a parameter *a *(Additional File 1 - Supplementary methods: *Network-based feature selection methods focusing on the network properties of individual genes - NetRank*).

We next considered the method by [8] (referred to for clarity herein as Taylor's method) based on identifying differential network behaviour within sub-networks. To perform feature selection the average difference in correlation (calculated for each edge in a network by considering the expression profiles of the protein-pair joined by the edge) for each hub sub-network over the two groups of interest was calculated. This calculation offered a measure of differential correlation for each hub sub-network which was used to select the most differentially correlated hub sub-networks. The edges from the most differentially correlated hub sub-networks were then used as the classification features and each edge was quantified by the expression difference between the hub gene and interactor gene joined by the edge (Additional File 1 - Supplementary methods: *Network-based feature selection methods focusing on sub-networks - Taylor's approach and BSS/WSS*).

Finally, we used the *BSS/WSS *approach, inspired by Taylor's differential correlation measure. However, instead of calculating the difference in correlation between the two prognostic groups of interest, we calculated the *BSS/WSS *ratio for the correlation values over those groups. This ratio was then used to rank the hub sub-networks from most to least differentially correlated and the hub genes from the hub sub-networks considered to be the most differentially correlated were then used as the classification features (Additional File 1 - Supplementary methods: *Sub-network-based feature selection methods for the network approach - Taylor's approach and BSS/WSS*).

**Evaluation criterion1 - stability: **We evaluated the stability of each feature selection method - single-gene, gene-set, and network-based - by considering the average number of selected features that were common to each pair of the 500 cross-validation (CV) folds (corresponding to the 100 rounds of 5-fold CV). We examined the average stability for each method when selecting the top 20, 30, 40 and 50 features in each of the CV folds.

**Evaluation criterion 2 - error estimation under 5-fold cross-validation: **For all models we performed 5-fold CV [54]*i.e*., splitting the data into five equal subsets (or as equal as possible). Here, one subset (referred to as the test set) was withheld for testing purposes while the remaining samples were used to train the classifier. The withheld subset was then used to estimate an error rate for the classifier. This procedure was repeated using each subset as the test set to obtain a total of five error rates. The 5-fold CV error rate was then defined to be the average of these five error rates. The final error rate estimate was defined to be the average 5-fold CV error rate over 100 rounds (Additional File 2 - Supplementary Figure 1).

**Evaluation criteria 3 - accuracy analysis at a patient-level: **To perform a detailed comparison of the relative performance of each method considered, we scrutinized the classification accuracy achieved for each individual patient. In each fold of the 5-fold CV procedure, the class of each sample in the test set was predicted. Since each sample appeared in the test set exactly once per round of 5-fold CV, we had exactly one class prediction for each patient following each round. Thus, we calculated patient-specific classification error rates by identifying the proportion of CV rounds in which the patient was misclassified (see Figure 5 legend for additional details).

## Results

### A comparison of single-gene, gene-set and network methods in melanoma identified NetRank as the most accurate method among them

**Figure 2:**
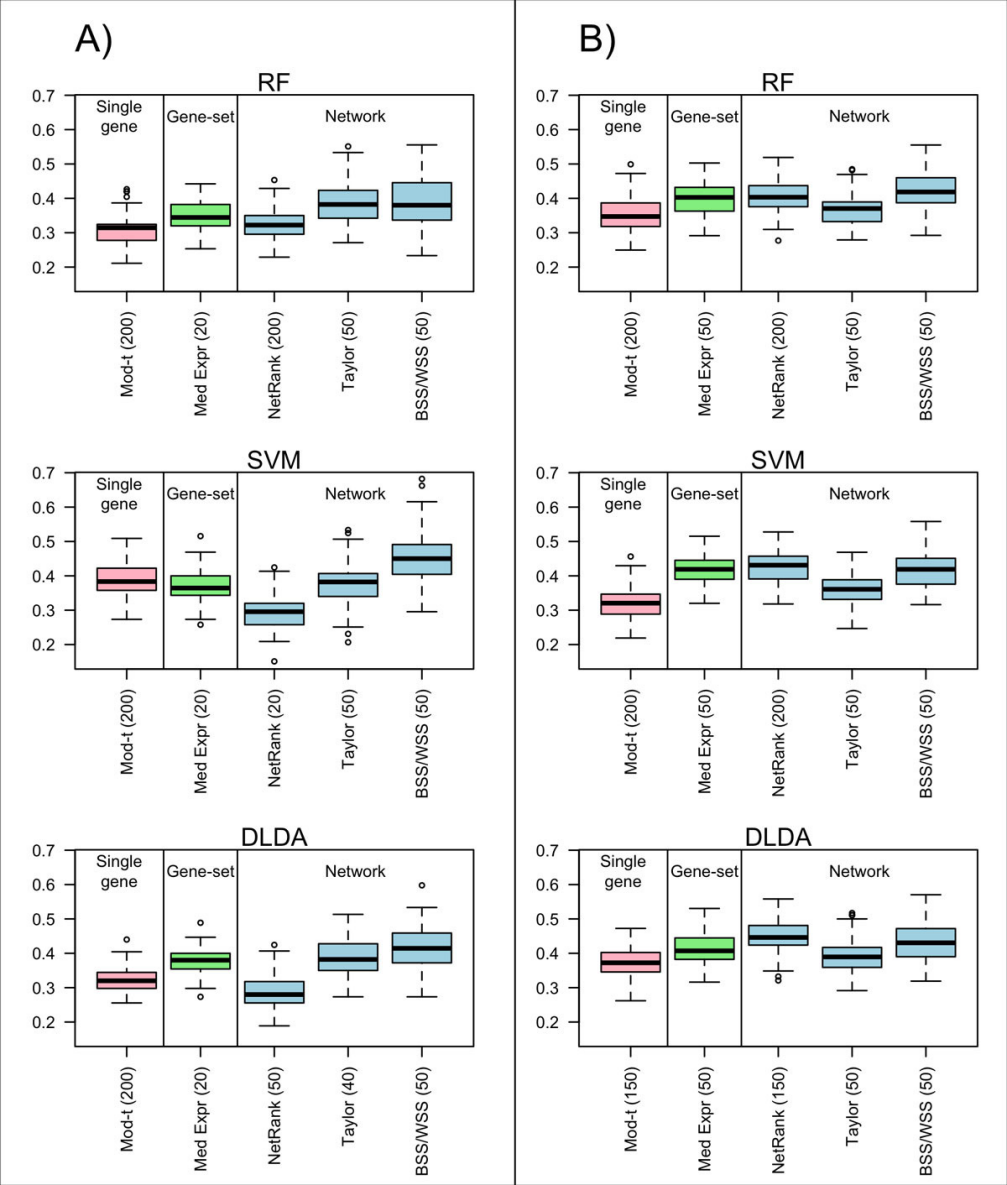
**Classification error rates**. The error rates (y-axis) obtained from 100 rounds of 5-fold cross validation are presented for the RF classifier, the SVM classifier and the DLDA classifier for iRefWeb network and A) the melanoma data set and B) the ovarian cancer data set. The numbers within the parentheses following the method names are the number of selected features in each cross-validation round.

For each method evaluated (Table 1), error rates were estimated using 100 rounds of 5-fold cross-validation for each of the RF, SVM, and DLDA classifiers. The 5-fold CV error rates achieved by the single-gene, gene-set and network methods for the melanoma data set and the iRefWeb network using the RF, SVM, and DLDA classifiers respectively, were: 31% (RF), 39% (SVM), and 33% (DLDA) for the single-gene moderated *t*-statistic method; 35% (RF), 37% (SVM), and 37% (DLDA) for the gene-set median expression method; 33% (RF), 29% (SVM), and 29% (DLDA) for the *NetRank *network-based method; 39% (RF), 38% (SVM), and 39% (DLDA) for Taylor's network method; and, 39% (RF), 45% (SVM), and 41% (DLDA) for the *BSS/WSS *network method (Figure 2A). The network-based *NetRank *method was thus the most accurate method, achieving the lowest error rates. This was followed by the single-gene moderated *t*-statistic method which performed similarly for the RF and DLDA classifiers but much poorer for the SVM classifier and, achieved an error rate that was 10% higher than that for *NetRank*. The gene-set median expression method performed slightly better than Taylor's network method, which was comparable to the single-gene moderated *t*-statistic method for the SVM classifier, but was much less accurate for the RF and DLDA classifiers.

### The same comparison of methods using ovarian cancer data did not confirm the relative accuracy of the NetRank approach

For the ovarian cancer data set, 5-fold CV error rates achieved by the single-gene, gene-set and network methods using the iRefWeb network and the RF, SVM and DLDA classifiers respectively, were: 35% (RF), 32% (SVM), and 37% (DLDA) for the single-gene moderated *t*-statistic method; 40% (RF), 42% (SVM), and 41% (DLDA) for the gene-set median expression method; 41% (RF), 43% (SVM), and 45% (DLDA) for the *NetRank *network-based method; 36% (RF), 36% (SVM), and 39% (DLDA) for Taylor's network method; and 42% (RF), 42% (SVM), and 43% (DLDA) for the *BSS/WSS *network method (Figure 2B). Compared with the observations made in melanoma above, the error rates for the ovarian cancer data were slightly higher and the *NetRank *network method no longer appeared to be the most accurate method. Instead, the single-gene moderated *t*-statistic methods performed best followed closely by Taylor's network method. The median expression gene-set method performed similarly to the *BSS/WSS *network method whereas the median expression gene-set method was more accurate than the *BSS/WSS *network method for the melanoma data set.

### Comparative analyses of within-class error revealed that the different classes achieved different error rates

**Figure 3:**
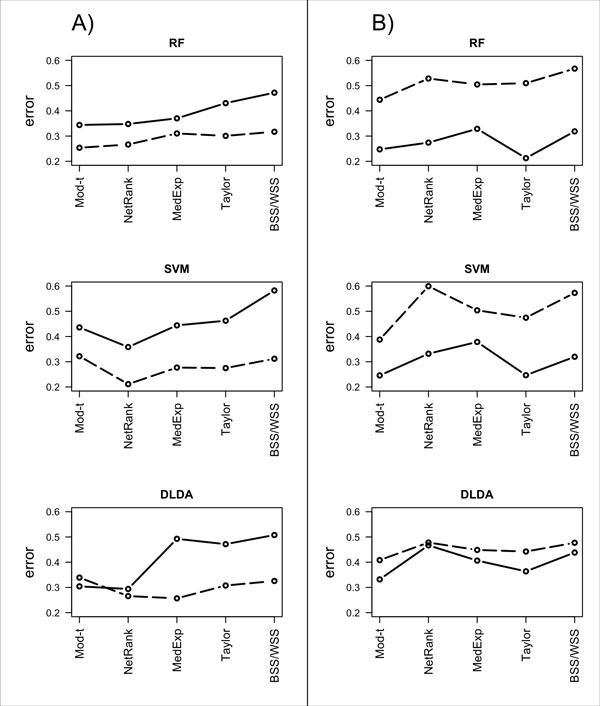
**Class-specific classification error rates**. The GP (dotted line) and PP (solid line) error rates averaged over the 100 rounds of 5-fold cross validation for each method are presented for the iRefWeb network and the RF classifier, the SVM classifier and the DLDA classifier using A) the melanoma data set and B) the ovarian cancer data set.

An evaluation of the class-specific (good versus poor prognosis) error rates for each of the methods revealed that patients with good prognosis were easier to classify than patients with poor prognosis in the melanoma data set (Figure 3A). Specifically, for the RF classifier error rates for all methods ranged from 34-47% for the PP class and from 25-32% for the GP class. Under SVM classification, error rates ranged from 36-58% for the PP class and from 21-32% for the GP class. Using the DLDA classifier, error rates ranged from 29-51% for the PP class and from 26-34% for the GP class. The only exception to this observation was in case of the single-gene moderated *t*-statistic and *NetRank *methods under the DLDA classifier in which the PP class and the GP class had similar classification error rates.

Similarly, error rates varied among survival classes in the ovarian cancer data. However, in these data, lower average error rates were observed for patients classified as PP (Figure 3B). For the RF classifier, the PP class achieved average error rates ranging from 21-33%, while the GP class achieved error rates ranging from 44-57%. For the SVM classifier, the PP class achieved error rates ranging from 25-38% and the GP class achieved error rates ranging from 39-60%. Finally, for the DLDA classifier, the PP class achieved error rates ranging from 33-47% and the GP class achieved error rates ranging from 41-48%. The difference in error rate between the PP class and the GP class was notably less extreme for the DLDA classifier.

### Comparative assessment of feature stability among single-gene, gene-set, and network-based approaches: NetRank performed best

**Figure 4:**
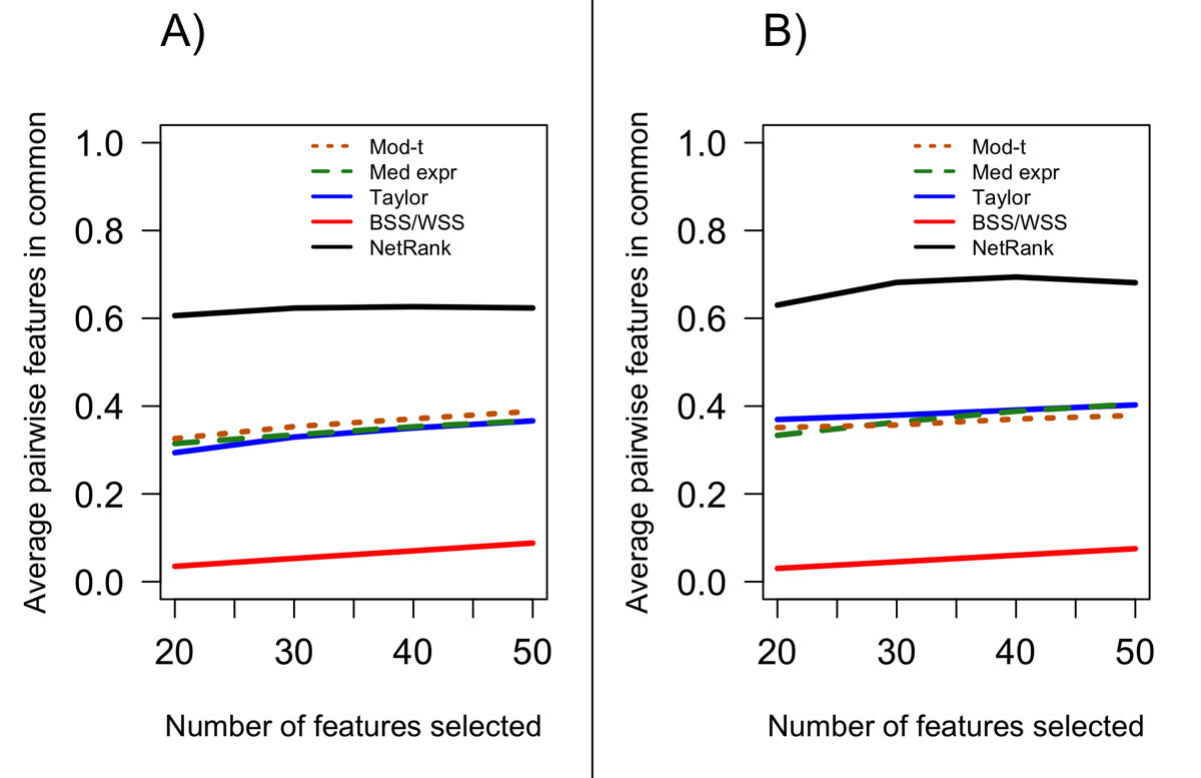
**Stability of the feature selection methods**. The number of selected features pair-wise in common over the 100 rounds of 5-fold cross-validation (thus over a total of 500 selected feature lists) for each of the single-gene, gene-set and network methods based on the iRefWeb PPI network for A) the melanoma data set and B) the ovarian cancer data set, with respect to the number of features selected.

The stability of the network-based *NetRank *method exceeded the stability of all other methods, with an average of 63% of features in common for the CV fold pairs when considering the top 50 features (Figure 4A). In this respect it out-performed the single-gene moderated *t*-statistic method which had very similar stability to Taylor's network-based method. *NetRank *was also more stable than the median-expression gene-set method. These methods each had an average of 38% of features in common for their CV fold pairs when considering the top 50 features. The *BSS/WSS *network-based method was the least stable with an average of only 9% of features in common among the CV fold pairs when considering the top 50 features. The relative stability of each feature selection method observed in the melanoma data set was comparable to those observed using the ovarian data (Figure 4B).

**Figure 5:**
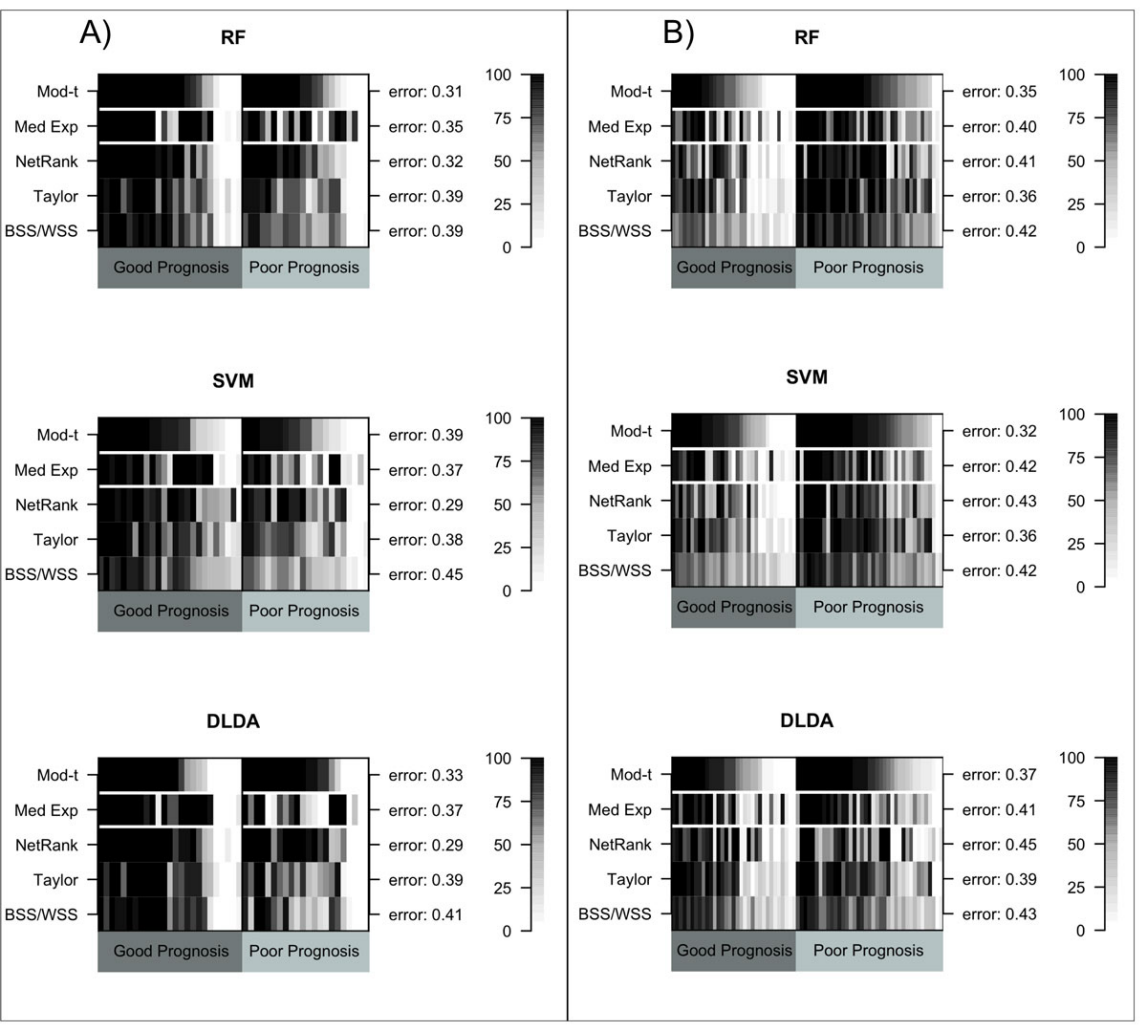
**Classification accuracy at the patient level**. A black cell corresponds to the patient being classified correctly in all 100 CV rounds, whereas a white cell corresponds to the patient being classified correctly in none of the 100 CV rounds for the RF classifier, the SVM classifier and the DLDA classifier using A) the melanoma data set and B) the ovarian cancer data set, together with the iRefWeb PPI network. The rows are split into single-gene (the first row), gene-set (the second row) and network-based method (the last three rows). The tumor IDs are given on the x-axis, and the average error rate (taken over the 100 rounds of CV) are provided on the right-hand-side y-axis.

### Single-gene, gene-set, and network-based methods captured different subspaces of the cohorts evaluated

We undertook a novel comparison of the single-gene, gene-set, and network-based approaches at the level of individual patients (Figure 5A, Additional Files 3,4,5). Overall, 10-15 samples in the melanoma dataset (depending on which classifier was used) were almost always classified correctly by every method. We refer to these henceforth as samples being 'easy to classify'. 7-9 samples (again, dependent on the classification algorithm) were almost never classified correctly by any method. Conversely, we refer to these samples as being 'hard to classify'. The remaining samples were better classified by some methods than by others.

Overall, the network-based *NetRank *method and the single-gene moderated *t*-statistic method performed similarly at the level of individual samples. In particular, they performed better than the gene-set median expression method, the *BSS/WSS *method, and Taylor's approach. We noticed that for the 10-14 PP samples which were very accurately classified by the single-gene moderated *t*-statistic method and the *NetRank *network-based method, the median expression gene-set and remaining network methods were much less accurate in classifying all but 3 of these samples. On the other hand, there were approximately 2-6 GP and 2-3 PP samples (depending on which classifier is used) which were more accurately classified by these gene-set and network methods *cf*. the single-gene moderated *t*-statistic method and the *NetRank *network-based method. In sum, the single-gene moderated *t*-statistic method and the *NetRank *network-based method captured a different subspace of the samples relative to the median expression gene-set method, Taylor's network method and the *BSS/WSS *network approach.

For the ovarian cancer data set, we observed 13-17 samples (classifier-dependent) that were almost always classified correctly by every method. Similarly, 9-11 samples were almost never classified correctly by any method (Figure 5B, Additional Files 6,7,8). Although the *NetRank *network method and the single-gene moderated *t*-statistic method appeared to perform similarly at the patient level for melanoma, this trend was not observed in ovarian cancer. Nonetheless, gene-set and network methods continued to capture different subsets of the sample space *cf*. the single-gene moderated *t*-statistic method. For example, the gene-set median expression method less accurately classified 8-9 samples but more accurately classified 5-7 samples. In contrast, *NetRank *less accurately classified 11-17 samples *cf*. the single-gene method but more accurately classified 4-11 samples. In addition, Taylor's method less accurately classified 7-9 samples compared with the single-gene method while more accurately classifying 7 samples. *BSS/WSS *less accurately captured 13-20 samples *cf*. the single-gene method and more accurately classified 1-4 samples.

### Observations relating to network-methods were validated using an independent PPI network

When we repeated the analysis in melanoma using the MetaCore™ PPI network in place of the iRefWeb PPI network the same general patterns were observed (Additional File 9: Supplementary results). That is: 1) the single-gene moderated *t*-statistic method and the network-based *NetRank *method performed slightly better in terms of classification error rate than the remaining methods (Additional File 10: Supplementary Figure 2); 2) the samples in the GP class were classified more accurately than the samples in the PP class (Additional File 11: Supplementary Figure 3); 3) *NetRank *was the most stable feature selection method *cf. BSS/WSS *which was the least stable while the remaining methods all displayed similar stability (Additional File 12: Supplementary Figure 4); and, 4) the various methods evaluated captured different subspaces of the sample space (Additional File 13: Supplementary Figure 5, Additional Files 14,15,16: Supplementary Tables 7-9).

## Discussion

Development of accurate prognostic gene expression signatures is a central challenge in clinical cancer research. We aimed to analyse and compare commonly employed single-gene and gene-set methods for prognostic classification alongside the more recent network-based approaches involving integration of PPI networks with gene expression information [8,11]. To focus our research on the potential value of these newer PPI network-based methods we excluded methods requiring additional information beyond *a priori *PPI knowledge. For the same reason, we also limited our comparison to the gene-set approach (median expression, [30,39]) which is a direct analog of the single-gene moderated-t statistic method [30] also considered herein. Moreover, and for the first time, we conducted an analysis at the level of individual patients as well as with respect to the classes being compared (good versus poor prognosis). We undertook our analyses using gene expression microarray information from two previously reported studies in separate cancers (melanoma [13] and ovarian [15]) and utilised PPI information from two different networks (MetaCore™ and iRefWeb [50]). Each of the methods analysed comprised a feature-selection algorithm which was followed by classification in R [53] using each of three different classifiers: RF, SVM and DLDA. All classification error rates were estimated using 100 rounds of 5-fold CV [54].

### Single-gene, gene-set, and network-based methods achieved similar error-rates, confirming prior observations and emphasizing questions about their value

Our first finding - that the different approaches achieved similar error rates with respect to each other - validates prior observations by [19-21]. Specifically, none of the classifiers employing composite features derived from secondary PPI data sources (the network-based approaches) out-performed the classical single-gene/gene-set approaches. We also observed that their performance in terms of error-rates varyied with the cancer data set being analysed. For example, *NetRank *achieved the lowest error rates overall in the melanoma cohort, a finding that did not hold up using the ovarian cancer data set but which was consistent with the findings of [55] who noted that of the 25 datasets they analysed, *NetRank *outperformed a number of classical single-gene methods in 23 of them.

### Patient-level analyses indicated there is value to be gained from network-based methods

Prior related reports have described and discussed the single-gene, gene-set, and network-based approaches to gene expression signature modelling to varying degrees [6,56]. However, these works did not analyse first hand nor consider in detail the methods for translation of such feature selection methods into a general classification framework. Of the previous studies that did undertake formal evaluations, the focus has been upon network- and pathway-based classifiers for outcome prediction in breast cancers [19-21] or on comparing the effect of using different kinds of external biological information in the learning process like functional annotations, PPIs, and expression correlation among genes [57]. Further, these previous studies draw their conclusions based primarily on classification error rates, whereas a particular novelty of this study is that we considered (for the first time) our results at the more in-depth level of individual patients. Within this framework, we observed that different methods were capturing different subsets of the sample space *i.e*., the different approaches were correctly classifying different samples. These results would suggest that new composite classification methods are needed to capture the complementary value of network-based, gene-set and classical single-gene approaches.

### Analyses of error rates within the good and poor survival classes further highlights the need for a composite approach to gene expression signature modelling

Our finding that samples from the GP class were easier to classify in melanoma, but samples from the PP class were easier to classify in ovarian cancer, highlights an underlying dataset dependency of these gene-set and network approaches. We also note some overall differences between the datasets. Performing a moderated *t*-statistic differential expression analysis on each data set (Additional File 9 - Supplementary results: *Comparison of the ovarian cancer data set and the melanoma data set*), we found that the melanoma data set contained 96 DE genes (*p*-value < 0.1) while the ovarian cancer data set had only 13 DE genes. This observation could offer an explanation as to why the methods focusing on identification of individual informative genes (the single-gene moderated *t*-statistic method and the *NetRank *network method) perform better on the melanoma data set than in the ovarian cancer data. Furthermore, there was no overlap between the 100 most DE genes from each data set. Using Taylor's differential correlation measure, and an arbitrary threshold of 0.5, we found that the melanoma data set contained 23 differentially correlation hub sub-networks and the ovarian cancer data set contained only 7. Thus in general it seems as though the melanoma data set contained more differential features between the PP and GP classes than the ovarian data set, which could explain the lower error rates obtained for melanoma.

### NetRank is the most stable approach, supporting the potential value of network-based approaches to prognostication

Our assessment of the stability of features identified in each method highlighted *NetRank *[11] as the most stable approach in both cancers. This finding is consistent with observations made by [55] who found that cancer-related signatures identified by *NetRank *had significant overlap between the data sets they considered. The study also showed that performing classification on datasets with the aim of classification into prognostic classes (as was the goal here) was notably less accurate than when the aim was to address diagnosis or sub-typing classification problems,. This finding again confirms that incorporation of network information may lead to identification of more stable gene expression signatures.

### Findings were validated in an independent PPI network, suggesting network invariance of the network methods

Our reproduction of findings obtained using the iRefWeb PPI network but instead using the MetaCore™ PPI network demonstrates that the network methods are not hugely dependent on the PPI network used. Strengthening this claim we note also that although the two networks contain many of the same proteins, they are somewhat different from one another in terms of global structure (Additional File 9 - Supplementary Results: *Comparison of the MetaCore™ and iRefWeb PPI networks*).

### Ongoing network issues

Network-based approaches continue to have important limitations of consequence to their translational relevance. In a PPI network, nodes correspond to protein-coding genes, and the existence of an edge between two such genes implies that the proteins coded for by those genes interact in a biological setting. One of the most significant issues in PPI network analysis is the inaccuracy and the lack of reliability of the available networks. It has been noted that of the huge number of currently available networks, there is very little overlap and consistency between them [58-61]. Currently, the two main approaches for identification of protein-protein interactions are the yeast two-hybrid (Y2H) and the affinity purification of complexes followed by mass spectrometry (AP-MS). Details on these methods can be found in [62]. Moreover, despite the huge number of publicly and privately available PPI databases, there is no interaction database covering the entire human genome. However, there are ongoing projects, such as the Human Interactome Project [63], with the aim of developing a complete human PPI map. Although our study showed that the network methods are not hugely dependent on the PPI network used, full elucidation of the extent to which the *a priori *information impacts the findings remains an open question.

## Conclusions

The contextualisation of high-throughput data sets using large-scale molecular interaction networks is emerging as an increasingly popular bioinformatics approach for the analysis of complex disease. Accurate prognostic information is essential for clinicians to be able to reliably stratify patients for a comparative assessment of therapeutic interventions. But what, if anything, do these next-generation methods add to traditional single-gene and gene-set approaches? Our comparative analysis reiterated that when assessed by error rates alone no single approach reliably out-performed any other. However, for the first time, we show that the different methods - single-gene, gene-set, and network-based - are correctly capturing different areas of the sample space. These findings imply that network approaches do indeed have the potential to enhance existing methods; we posit, for example, through development of 'combination'-type classifiers that are capable of identifying the subset of patients for whom one approach may be more accurate compared with another.

## Availability of supporting data

The data sets supporting the results of this article are available in the Gene Expression Omnibus (GEO) repository: GSE53118 for melanoma [13] and GSE26712 for ovarian cancer [15].

## List of abbreviations

AP-MS affinity purification mass spectrometry

BSS between sum-of-squares

CV cross-validation 

DE differentially expressed

DLDA diagonal linear discriminant analysis

GAGE generally applicable gene-set enrichment

GP good prognosis

GSA gene-set analysis

GSEA gene-set enrichment analysis

PP poor prognosis

PPI protein-protein interaction

RF random forest

SVM support vector machines

WGCNA weighted gene co-expression network analysis

WSS within sum-of-squares

Y2H yeast two-hybrid

## Competing interests

The authors declare that they have no competing interests.

## Authors' contributions

YHY conceived of the study. RB carried out the analysis in R, supervised by YHY. All authors participated in the design of the study and made substantial contributions to the analysis and interpretation of data. RB and SJS performed the literature review, drafted the manuscript and associated supplements, and prepared the figures and tables. All authors reviewed, critically analysed, and approved the final manuscript.

## Supplementary Material

Additional File 1 Supplementary methods. Mathematical descriptions of the methods including the single-gene moderated *t*-statistic approach, the median expression gene-set approach, the network-based NetRank approach, Taylor's network-based approach and the BSS/WSS network-based approach. Click here for file

Additional File 2 **Supplementary Figure 1**. A flow chart showing the general method for performing 5-fold cross-validation. Click here for file

Additional File 3 **Supplementary Table 1**. A numerical representation of the number of cross-validation rounds (out of 100) in which each sample is correctly classified when using the melanoma dataset with the iRefWeb PPI and the RF classifier. The samples are identified by their Tumour IDs. The order of the samples are the same as presented in Figure 5A) RF. Click here for file

Additional File 4 **Supplementary Table 2**. A numerical representation of the number of cross-validation rounds (out of 100) in which each sample is correctly classified when using the melanoma dataset with the iRefWeb PPI and the SVM classifier. The samples are identified by their Tumour IDs. The order of the samples are the same as presented in Figure 5A) SVM. Click here for file

Additional File 5 **Supplementary Table 3**. A numerical representation of the number of cross-validation rounds (out of 100) in which each sample is correctly classified when using the melanoma dataset with the iRefWeb PPI and the DLDA classifier. The samples are identified by their Tumour IDs. The order of the samples are the same as presented in Figure 5A) DLDA. Click here for file

Additional File 6 **Supplementary Table 4**. A numerical representation of the number of cross-validation rounds (out of 100) in which each sample is correctly classified when using the ovarian cancer dataset with the iRefWeb PPI and the RF classifier. The samples are identified by their Tumour IDs. The order of the samples are the same as presented in Figure 5B) RF. Click here for file

Additional File 7 **Supplementary Table 5**. A numerical representation of the number of cross-validation rounds (out of 100) in which each sample is correctly classified when using the ovarian cancer dataset with the iRefWeb PPI and the SVM classifier. The samples are identified by their Tumour IDs. The order of the samples are the same as presented in Figure 5B) SVM. Click here for file

Additional File 8 **Supplementary Table 6**. A numerical representation of the number of cross-validation rounds (out of 100) in which each sample is correctly classified when using the ovarian cancer dataset with the iRefWeb PPI and the DLDA classifier. The samples are identified by their Tumour IDs. The order of the samples are the same as presented in Figure 5B) DLDA. Click here for file

Additional File 9 **Supplementary results**. Results based on the MetaCore™ PPI network and comparisons of the MetaCore™ network with the iRefWeb network and the ovarian cancer dataset with the melanoma dataset. Click here for file

Additional File 10 **Supplementary Figure 2**. Classification error rates obtained from 100 rounds of 5-fold cross-validation for the melanoma dataset and MetaCore™ PPI network. The error rates are presented for the RF classifier, the SVM classifier and the DLDA classifier for the melanoma dataset and the MetaCore™ PPI network. The numbers within the parentheses following the method names are the number of selected features in each cross-validation round. Click here for file

Additional File 11 **Supplementary Figure 3**. Class-specific classification error rates obtained from the average of 100 rounds of 5-fold cross validation for the melanoma dataset and MetaCore™ PPI network. The average GP (dotted line) and PP (solid line) error rates for each method are presented for the RF classifier, the SVM classifier and the DLDA classifier using the melanoma dataset and the MetaCore™ PPI network. Click here for file

Additional File 12 Supplementary Figure 4. Stability for the feature selection methods for the melanoma dataset and MetaCore™ PPI network. The number of selected features pair-wise in common over the 100 rounds of 5-fold cross-validation (thus over a total of 500 selected feature lists) for each of the single-gene, gene-set and network methods based on the MetaCore™ PPI network for the melanoma dataset, with respect to the number of features selected. Click here for file

Additional File 13 Supplementary Figure 5. Classification accuracy at the patient level for the melanoma dataset and the MetaCore™ PPI network. A black cell corresponds to the patient being classified correctly in all 100 CV rounds, whereas a white cell corresponds to the patient being classified correctly in none of the 100 CV rounds for the RF classifier, the SVM classifier and the DLDA classifier using the MetaCore™ PPI network and the melanoma dataset. The rows are split into non-grouping methods (first two rows) and grouping methods (last three rows). The tumor IDs are given on the x-axis, and the average error rate (taken over the 100 rounds of CV) are provided on the y-axis on the right-hand side. Click here for file

Additional File 14 **Supplementary Table 7**. A numerical representation of the number of cross-validation rounds (out of 100) in which each sample is correctly classified when using the melanoma dataset with the MetaCore™ PPI network and the RF classifier. The samples are identified by their Tumour IDs. The order of the samples are the same as presented in Supplementary Figure 5) RF. Click here for file

Additional File 15 **Supplementary Table 8**. A numerical representation of the number of cross-validation rounds (out of 100) in which each sample is correctly classified when using the melanoma dataset with the MetaCore™ PPI network and the SVM classifier. The samples are identified by their Tumour IDs. The order of the samples are the same as presented in Supplementary Figure 5) SVM. Click here for file

Additional File 16 **Supplementary Table 9**. A numerical representation of the number of cross-validation rounds (out of 100) in which each sample is correctly classified when using the melanoma dataset with the MetaCore™ PPI network and the DLDA classifier. The samples are identified by their Tumour IDs. The order of the samples are the same as presented in Supplementary Figure 5) DLDA. Click here for file

## Declarations

Publication costs for this article were funded by an Australian Research Council Future Fellowship (DP130100488) to YHY.

This article has been published as part of *BMC Systems Biology *Volume 8 Supplement 4, 2014: Thirteenth International Conference on Bioinformatics (InCoB2014): Systems Biology. The full contents of the supplement are available online at http://www.biomedcentral.com/bmcsystbiol/supplements/8/S4.
