# Supplementary material for: Network-based biomarkers enhance classical approaches to prognostic gene expression signatures
Source: BMC Syst Biol. 2014 Dec 8;8(Suppl 4):S5. doi: 10.1186/1752-0509-8-S4-S5 (PMC4290694; doi:10.1186/1752-0509-8-S4-S5)
Supplement: Additional File 1 — Supplementary methods. Mathematical descriptions of the methods including the single-gene moderated t-statistic approach, the median expression gene-set approach, the network-based NetRank approach, Taylor's network-based approach and the BSS/WSS network-based approach. [file 1752-0509-8-S4-S5-S1.pdf]

Supplementary methods

Single-gene approaches – moderated t-statistic

The moderated *t*-statistic method is implemented via the lmFit and eBayes functions from the limma package [1] in R. A robust linear model is fit to the expression profiles for each gene, which can be represented as:

$$E(\mathbf{y}_j) = \mathbf{X}\beta_j \qquad (1),$$

where $\mathbf{y}_j$ is a vector of expression data for gene j, β_j is a vector of coefficients, and $\mathbf{X}$ is the design matrix. For each gene, a moderated *t*-statistic is calculated based on the above model, which has the same interpretation as an ordinary *t*-statistic, however the standard errors have been moderated across genes (shrunk towards a common value, using a Bayesian model). A corresponding FDR-adjusted *p*-value is then calculated for each gene.

The resultant *p*-values offer a measure of differential expression. The genes are ranked according to their *p*-values, with the top-ranked gene having the smallest *p*-value and thus being the most differentially expressed. The m most differentially expressed genes are then selected to be the classification features (we use $m=50, 100, 150, 200$) for the random forest (RF) [2], support vector machine (SVM) [3], and diagonal linear discriminant analysis (DLDA) [4] classifiers, each of which yields a class prediction for each patient.

Gene-set approaches – median expression

Instead of fitting a linear model to each gene, as in the single-gene moderated *t*-statistic method above, we fit a linear model to each gene-set (quantified by the median gene expression value of all genes in the set) and then calculate moderated *t*-statistics and the corresponding *p*-values [1]. The m gene-sets with the smallest *p*-values (most differentially expressed) are used as the classification features for the three classifiers (we use $m = 20, 30, 40, 50$).

Network-based feature selection methods focusing on the network properties of individual genes – NetRank

The NetRank algorithm [5] assigns a rank to each gene, which depends both on the rank of all genes connected to it via an edge in the network, and on the correlation of the gene's expression profile with survival time. The iterative ranking procedure is given by:

$$r_j^n = (1-a)c_j + a\sum_{i=1}^{M}\frac{w_{ij}r_i^{n-1}}{d(i)} \text{ for } 1 \le j \le M \qquad (2),$$

where r_j^n denotes the rank of gene *j* after *n* iterations, *M* is the total number of genes in the network, $W \in R^{M\times M}$ is a symmetric adjacency matrix for the network, c_j is the absolute correlation between the expression profile for gene *j* and patient survival time, $d(i)$ is the degree (number of edges incident) of gene *i,* and $a \in (0,1)$ is a fixed parameter describing the influence of the network on the gene ranking. We use the value *a*=0.85 after noting that this is the value used by both Google and Winter *et al.* and the results are quite stable with respect to adjustments of the value of *a*.

From the ranked list of genes obtained from the NetRank algorithm, we select the *m* top-ranked genes for classification (we use *m=20, 30, 40, 50, 100, 150, 200*).

Network-based feature selection methods focusing on sub-networks – Taylor's approach and BSS/WSS

The previous methods have all simply used gene expression (or median gene-set expression) as the classification feature values. When our features are sub-networks (or can be obtained from sub-networks), the situation is a bit more complicated. Given that our focus is on the topologically simple hub sub-networks, many network properties such as connectivity, modularity and node-node distance become irrelevant. As a result, most hub sub-network measures will be based on correlation between gene-expression values for pairs of genes connected by an edge in an attempt to identify differentially correlated sub-networks.

Unfortunately, it can be difficult to translate a correlation-based network measure into a classification framework due to the fact that correlation calculations require values from more than one patient. Thus a correlation-based measure cannot yield a unique vector of feature values for each patient, as is required for classification.

We will see that, for this reason, the methods described below will use correlation-based network measures to perform feature selection, but will use different feature types (such as network the edges or the hub genes in the selected networks) for classification.

The method described by [6] involves first determining which hub sub-networks are differentially correlated between the two classes, and then using the edges from the most differentially correlated sub-networks as the classification features. Taylor's method was the first to utilise the hub sub-network structures in a prognostic classification framework.

The correlation between a hub gene and an interactor gene is defined to be the Pearson correlation between their expression profiles across the patients. The difference between the correlation (Δ*C*) for each edge (hub-interactor pair), *i*, in a hub sub-network for the two classes of patients (PP and GP), is thus defined by:

$$\Delta C_{P,G,i} = \left(\frac{\sum_{k \in P} (I_k - \bar{I}_P)(H_k - \bar{H}_P)}{(n_P - 1) S_{I_P} S_{H_P}} \right) - \left(\frac{\sum_{k \in G} (I_k - \bar{I}_G)(H_k - \bar{H}_G)}{(n_G - 1) S_{I_G} S_{H_G}} \right) \qquad (3),$$

where, $\sum_{k \in P}$ Implies the sum is over the PP patients and $\sum_{k \in G}$ implies the sum is over the GP patients, I_k and H_k denote the gene expression values for the interactor gene and hub gene respectively, for the *k*th patient, n_P and n_G are the number of patients in the PP class and the GP class respectively, $\bar{I}_P$ and $\bar{H}_P$ are the average expression values over the PP patients for the interactor gene and the hub gene respectively, $\bar{I}_G$ and $\bar{H}_G$ are the average expression values over the GP patients for the interactor gene and the hub gene respectively, and $S_{I_P} S_{H_P}$ and

$S_{I_G} S_{H_G}$ are the products of standard deviations for the hub and interactor expression for PP and GP patients, respectively.

The network measure used to quantify differential correlation of a hub sub-network is defined to be the average absolute correlation difference over all edges in the hub sub-network, given by:

$$AveHubDif = \frac{\sum_{i=1}^{n} \left| \Delta C_{P,G,i} \right|}{n-1} \quad (4),$$

where *n* is the number of interactors/edges in the hub sub-network. This *AveHubDif* measure can be interpreted as a quantification of the extent to which the given hub sub-network is differentially correlated between the PP and the GP class. We then select the *m* hub sub-networks with the largest *AveHubDif* values for classification (Taylor *et al.* instead used a computationally intensive non-parametric test to determine if the difference in correlation was significant).

Since the calculation of the *AveHubDif* measure for any given hub sub-network used information from all samples, it is impossible to calculate a unique *AveHubDif* value for each individual patient, as is required for classification. For this reason, instead of using these hub sub-networks as the classification features, Taylor *et al.*'s method uses the edges within these top-ranked hub sub-networks. The feature value assigned to these edges for classification is equal to the expression difference between the hub gene and interactor gene joined by the edge:

$$InteractionDif_j = I_j - H \quad (5),$$

where I_j is the expression of the jth interactor gene in the hub sub-network, and *H* is the expression of the hub gene.

In a similar manner to that recapitulated above [6], the between-to-within sum-of-squares (BSS/WSS) method uses a correlation-based network measure to rank the hub sub-networks in order to perform feature selection. However,

since (as discussed above) such a network measure cannot be translated into a classification framework, we take the hub genes from the *m* top-ranked hub sub-networks to be the features for classification (we use *m=20, 30, 40, 50*).

In order to define our BSS/WSS network measure, we will first provide some notation. Suppose that for a given hub sub-network with *n* edges, C_{ij} is the edge correlation between the expression values of the jth interactor and the hub gene taken over all such pairs in the ith class (*i = PP* or *GP*). We then define:

$$\overline{C}_{i\bullet} = \frac{C_{i,1} + C_{i,2} + \cdots + C_{i,n}}{n} \quad (6),$$

to be the average edge correlation in the hub sub-network over the *i*th class and;

$$\overline{C}_{\bullet\bullet} = \frac{\overline{C}_{PP\bullet} + \overline{C}_{GP\bullet}}{2} \quad (7),$$

to be the average edge correlation for the hub sub-network over all samples.

The between sum-of-squares (*BSS*) and the within sum-of-squares (*WSS*) are then defined as follows:

$$BSS = \sum_{i=PP,GP} n(\overline{C}_{i\bullet} - \overline{C}_{\bullet\bullet})^2 \quad (8),$$

and;

$$WSS = \sum_{i=PP,GP} \sum_{j=1}^{n} (C_{ij} - \overline{C}_{i\bullet})^2 \quad (9).$$

BSS measures the variation between the average correlation within the PP group and the average correlation within the GP group (where correlation is calculated between the expression profiles over the PP or the GP patients for

each pair of genes joined by an edge in the hub sub-network). *WSS* measures the variation in correlation within the PP group and within the GP group. The hub sub-network measure is then defined to be the ratio:

$$\frac{BSS}{WSS} \qquad (10),$$

and the hub sub-networks are ranked such that the top-ranked hub sub-network has the largest *BSS/WSS* ratio. The *m* hub genes obtained from the *m* top-ranked hub sub-networks are then used as the features for classification (we use *m=20, 30, 40, 50*).

References

1. Smyth GK: **limma: Linear Models for Microarray Data.** In *Bioinformatics and Computational Biology Solutions Using R and Bioconductor.* Edited by Gentleman R, Carey V, Huber W, Irizarry R, Dudoit S: Springer New York; 2005: 397-420: *Statistics for Biology and Health*.
2. Breiman L: **Random Forests.** *Machine Learning* 2001, **45:**5-32.
3. Fan R-E, Chen P-H, Lin C-J: **Working Set Selection Using Second Order Information for Training Support Vector Machines.** *The Journal of Machine Learning Research* 2005, **6:**1889-1918.
4. Dettling M, Buhlmann P: **Supervised clustering of genes.** *Genome Biology* 2002, **3:**research0069.0061-research0069.0015.
5. Winter C, Kristiansen G, Kersting S, Roy J, Aust D, Knösel T, Rümmele P, Jahnke B, Hentrich V, Rückert F, et al: **Google Goes Cancer: Improving Outcome Prediction for Cancer Patients by Network-Based Ranking of Marker Genes.** *PLoS Computational Biology* 2012, **8:**e1002511.
6. Taylor IW, Linding R, Warde-Farley D, Liu Y, Pesquita C, Faria D, Bull S, Pawson T, Morris Q, Wrana JL: **Dynamic modularity in protein interaction networks predicts breast cancer outcome.** *Nature Biotechnology* 2009, **27:**199-204.
