# Supplementary figures and images for: Network-based biomarkers enhance classical approaches to prognostic gene expression signatures
Source: BMC Syst Biol. 2014 Dec 8;8(Suppl 4):S5. doi: 10.1186/1752-0509-8-S4-S5 (PMC4290694; doi:10.1186/1752-0509-8-S4-S5)

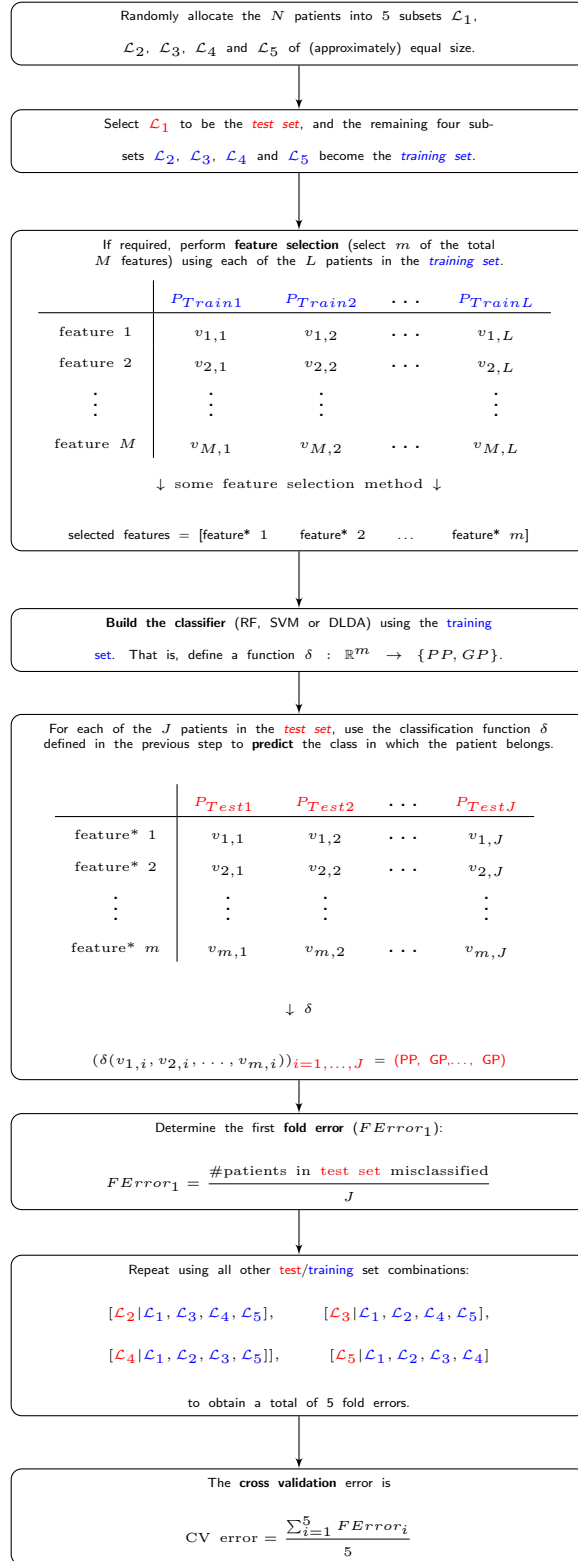

Supplement: Additional File 2 — Supplementary Figure 1. A flow chart showing the general method for performing 5-fold cross-validation. [file 1752-0509-8-S4-S5-S2.pdf]

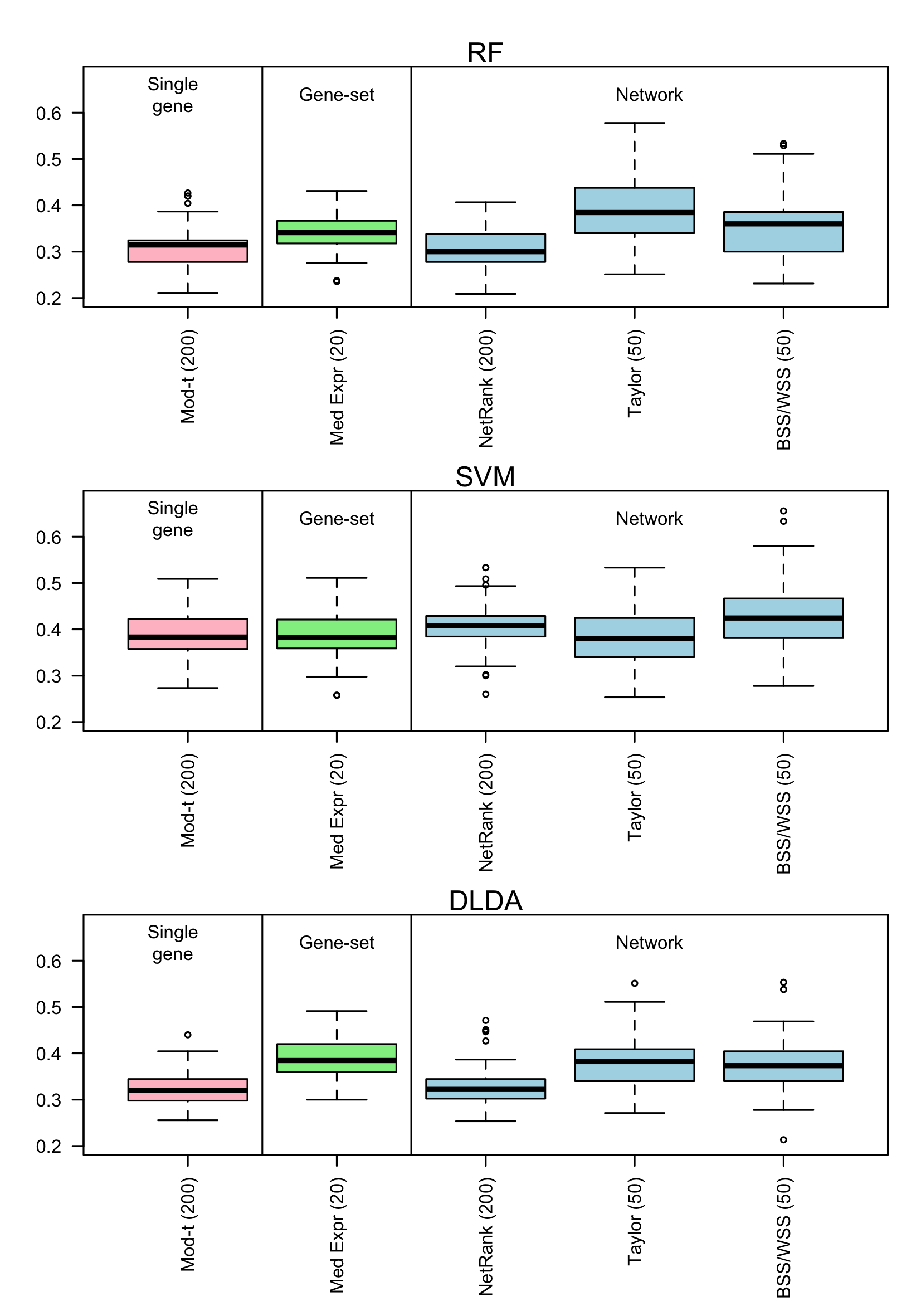

Supplement: Additional File 10 — Supplementary Figure 2. Classification error rates obtained from 100 rounds of 5-fold cross-validation for the melanoma dataset and MetaCore™ PPI network. The error rates are presented for the RF classifier, the SVM classifier and the DLDA classifier for the melanoma dataset and the MetaCore™ PPI network. The numbers within the parentheses following the method names are the number of selected features in each cross-validation round. [file 1752-0509-8-S4-S5-S10.png]

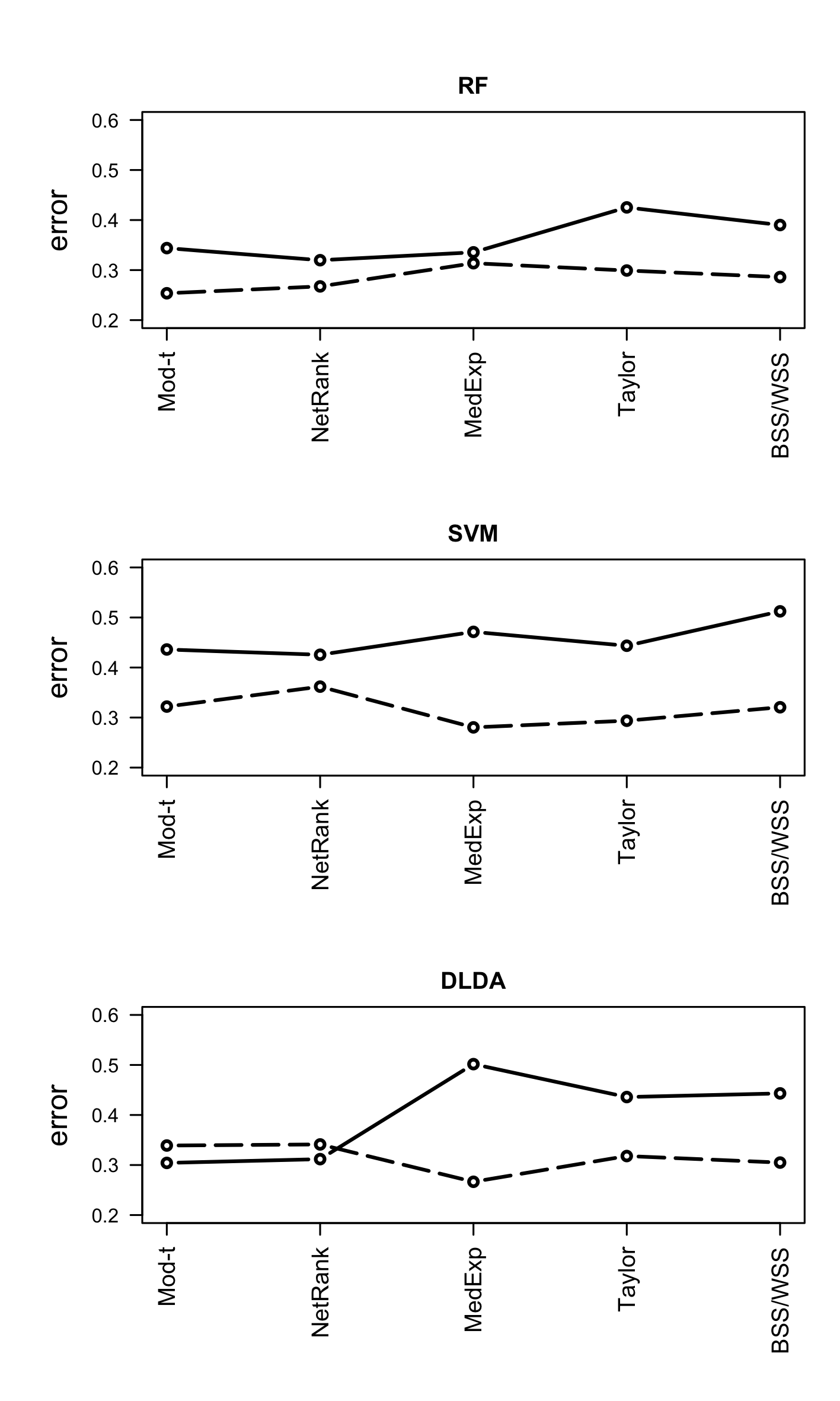

Supplement: Additional File 11 — Supplementary Figure 3. Class-specific classification error rates obtained from the average of 100 rounds of 5-fold cross validation for the melanoma dataset and MetaCore™ PPI network. The average GP (dotted line) and PP (solid line) error rates for each method are presented for the RF classifier, the SVM classifier and the DLDA classifier using the melanoma dataset and the MetaCore™ PPI network. [file 1752-0509-8-S4-S5-S11.png]

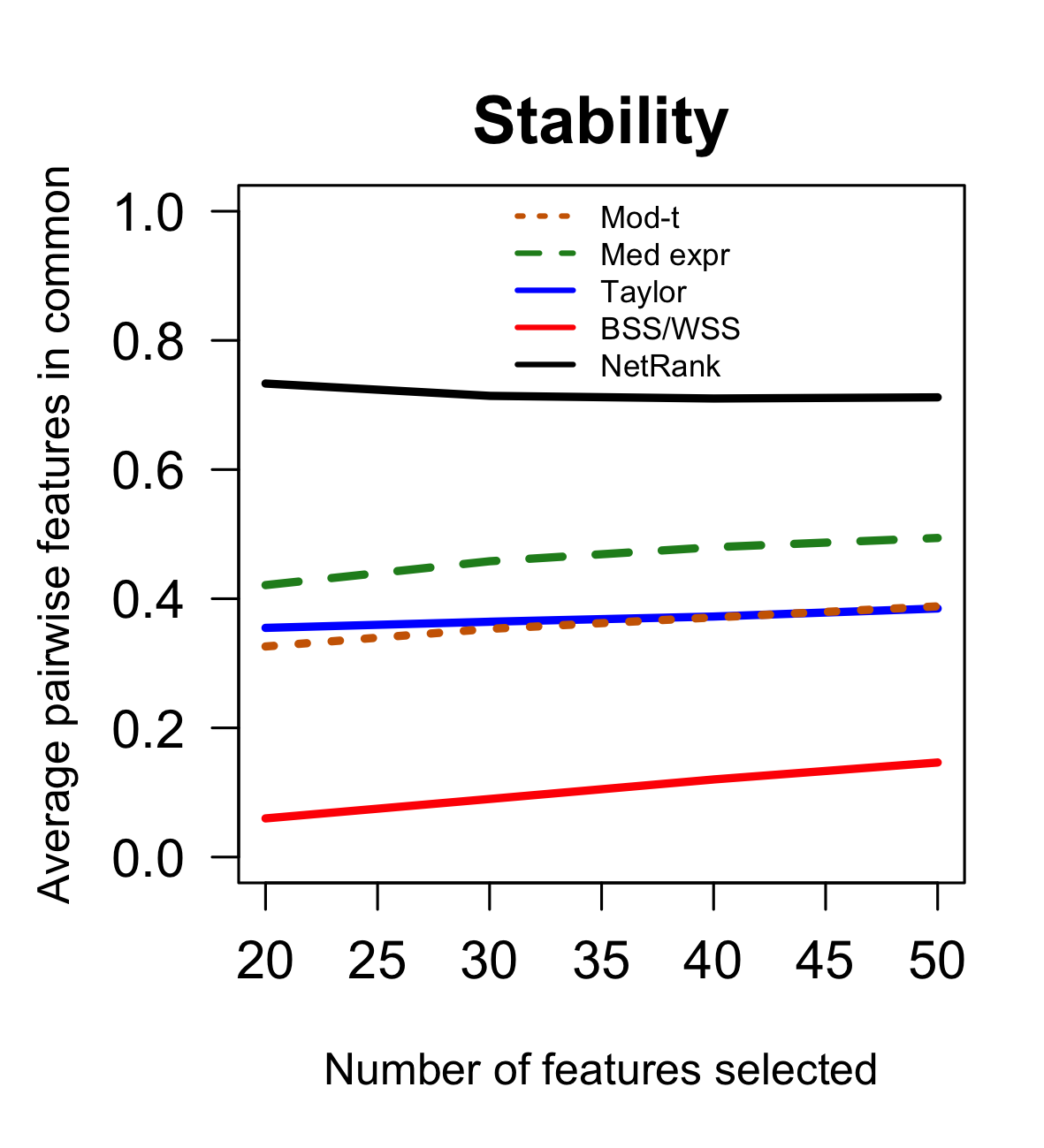

Supplement: Additional File 12 — Supplementary Figure 4. Stability for the feature selection methods for the melanoma dataset and MetaCore™ PPI network. The number of selected features pair-wise in common over the 100 rounds of 5-fold cross-validation (thus over a total of 500 selected feature lists) for each of the single-gene, gene-set and network methods based on the MetaCore™ PPI network for the melanoma dataset, with respect to the number of features selected. [file 1752-0509-8-S4-S5-S12.png]

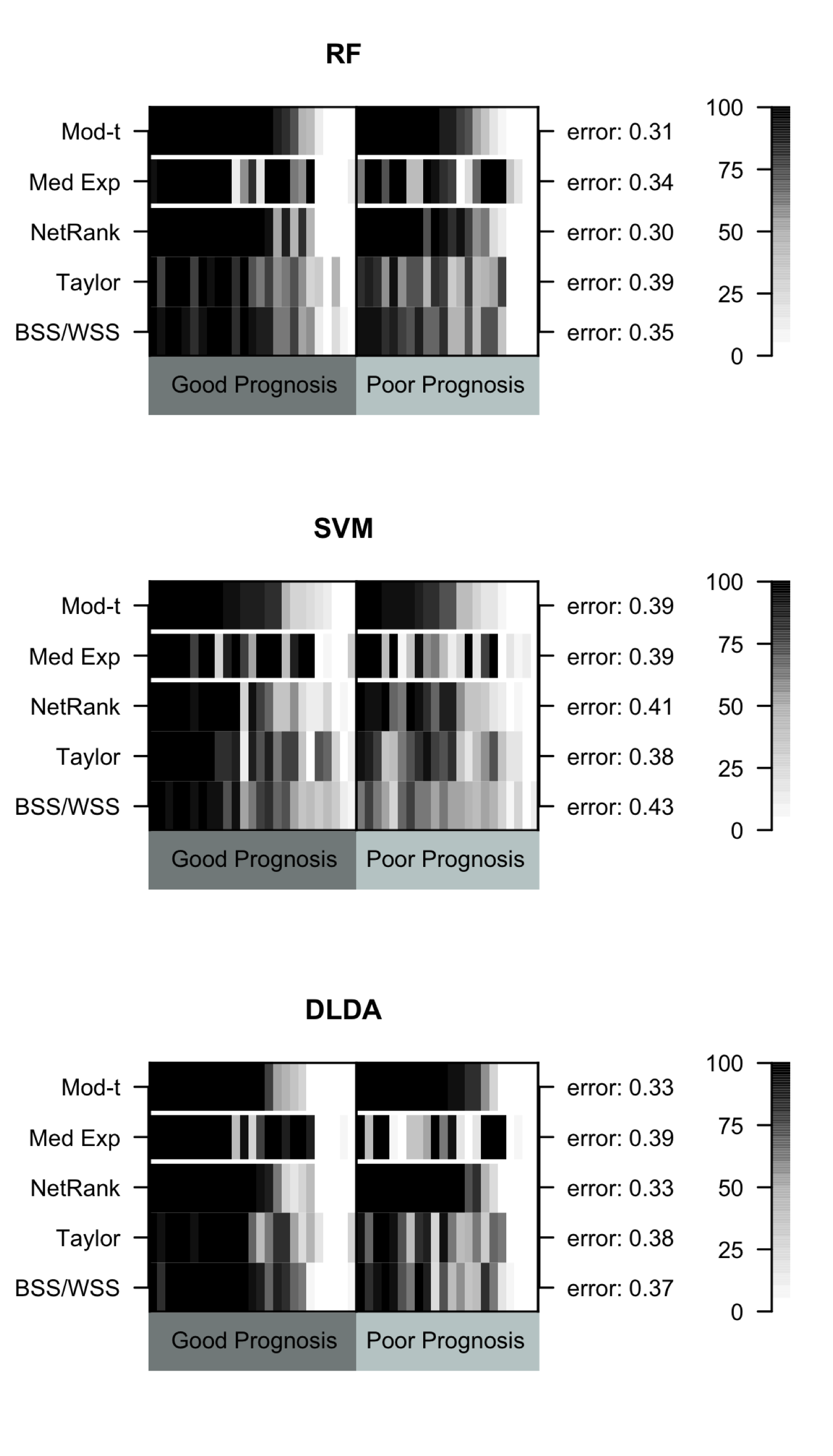

Supplement: Additional File 13 — Supplementary Figure 5. Classification accuracy at the patient level for the melanoma dataset and the MetaCore™ PPI network. A black cell corresponds to the patient being classified correctly in all 100 CV rounds, whereas a white cell corresponds to the patient being classified correctly in none of the 100 CV rounds for the RF classifier, the SVM classifier and the DLDA classifier using the MetaCore™ PPI network and the melanoma dataset. The rows are split into non-grouping methods (first two rows) and grouping methods (last three rows). The tumor IDs are given on the x-axis, and the average error rate (taken over the 100 rounds of CV) are provided on the y-axis on the right-hand side. [file 1752-0509-8-S4-S5-S13.png]
