# Supplementary material for: Network-based biomarkers enhance classical approaches to prognostic gene expression signatures
Source: BMC Syst Biol. 2014 Dec 8;8(Suppl 4):S5. doi: 10.1186/1752-0509-8-S4-S5 (PMC4290694; doi:10.1186/1752-0509-8-S4-S5)
Supplement: Additional File 9 — Supplementary results. Results based on the MetaCore™ PPI network and comparisons of the MetaCore™ network with the iRefWeb network and the ovarian cancer dataset with the melanoma dataset. [file 1752-0509-8-S4-S5-S9.pdf]

Supplementary Results

Classification using the MetaCore™ PPI - Comparison of single-gene, gene-set and network methods

For each method, error rates were estimated using 100 rounds of 5-fold cross-validation (CV) for each of the Random Forest (RF), linear Support Vector Machine (SVM) and Diagonal Linear Discriminant Analysis (DLDA) classifiers. The 5-fold CV error rates achieved by the single-gene, gene-set and network methods for the melanoma dataset and the MetaCore™ network using the RF, SVM and DLDA classifiers respectively, were 31% (RF), 39% (SVM) and 33% (DLDA) for the single-gene moderated *t*-statistic method, 34% (RF), 39% (SVM) and 39% (DLDA) for the gene-set median expression method, 30% (RF), 41% (SVM) and 33% (DLDA) for the NetRank network-based method, 39% (RF), 38% (SVM) and 38% (DLDA) for Taylor's network method and 35% (RF), 43% (SVM) and 37% (DLDA) for the BSS/WSS network method (Supplementary Figure 2). The network-based NetRank method performed very similarly to the single-gene moderated *t*-statistic method. The gene-set median expression method performed slightly better than Taylor's network method, which is comparable to the single-gene moderated *t*-statistic method for the SVM classifier, but is much less accurate for the RF and DLDA classifiers.

Classification using the MetaCore™ PPI - Comparison of the error rates achieved by the PP and GP classes

An evaluation of the class-specific error rates for each of the methods revealed that samples with a good prognosis are much easier to classify than samples with a poor prognosis in the melanoma dataset (Supplementary Figure 3). In particular, for the RF classifier the error rates of all methods considered ranged from 32-43% for the PP class and from 25-31% for the GP class; for the SVM classifier the error rates ranged from 43-51% for the PP class and from 28-36% for the GP class; and for the DLDA classifier the error rates ranged from 30-50% for the PP class and from 27-34% for the GP class. We note that the only exception to this observation is for the single-gene moderated *t*-

statistic and NetRank methods when using the DLDA classifier in which the PP class and the GP class have similar classification error rates.

Classification using the MetaCore™ PPI – Evaluation of stability

The stability of the network-based NetRank method (with an average of 71% of features in common for the CV fold pairs when considering the top 50 features) exceeded the stability of all other methods (Supplementary Figure 4), including the single-gene moderated *t*-statistic method (with an average of 39% of features in common for the CV fold pairs when considering the top 50 features), which has very similar stability to Taylor's network-based method (with an average of 39% of features in common for the CV fold pairs when considering the top 50 features), and is slightly less stable than the median-expression gene-set method (with an average of 49% of features in common for the CV fold pairs when considering the top 50 features). The BSS/WSS network-based method, however, was the least stable (with an average of only 15% of features in common for the CV fold pairs when considering the top 50 features).

Classification using the MetaCore™ PPI - The methods capture different subspaces of the sample

We undertook a patient-based comparison of the methods analysed (Supplementary Figure 5, Supplementary Tables 7-9). To begin with, 11-15 samples (depending on which classifier is used) were almost always classified correctly by every method (these samples are "easy to classify") and 8-9 samples (depending on which classifier is used) were almost never classified correctly by any method (these samples are "hard to classify"). The remaining samples are better classified by some methods than by others.

Overall, the network-based NetRank method and the single-gene moderated *t*-statistic method performed similarly at the level of individual samples, particularly in comparison to the performance the gene-set median expression method and the BSS/WSS and Taylor's network methods. More specifically, NetRank and the moderated *t*-statistic method are classifying 40-46 of the 47 total samples with similar accuracy, while the median expression gene-set

method is classifying 6-9 samples more accurately than the single-gene method and 10-13 samples less accurately. Taylor's method is classifying 1-5 samples more accurately than the single-gene method and 10-18 samples less accurately. Finally, the BSS/WSS method is classifying 0-2 samples more accurately than the single-gene method and 10-15 samples less accurately. Thus, we are seeing that different methods are correctly capturing different subsets of the sample space. This phenomenon is most obvious when comparing the median expression gene-set method with the single-gene moderated *t*-statistic method and the NetRank network method. We also note that Taylor's network method and the BSS/WSS network method performed particularly poorly on the PP samples.

Comparison of the MetaCore™ and iRefWeb PPI networks

Having observed very similar results when using the MetaCore™ PPI network in place of the iRefWeb PPI network, we offer a comparative analysis of the two networks to illustrate that they do not simply contain the same information. The following comparative analyses of the MetaCore™ and iRefWeb networks were performed using the igraph package [1] in R [2]. The iRefWeb network contains 7,256 nodes and 42,096 edges and is somewhat larger than the MetaCore™ network, which has 5,009 nodes and 32,404 edges. 3,313 of the nodes were common to both networks (corresponding 66% of the nodes in MetaCore™ and 46% of the nodes in iRefWeb) and only 4,754 of the edges were common to both networks (corresponding to 15% of the edges in MetaCore™ and 11% of the edges in iRefWeb). The networks have similar densities (defined by the number of edges in the network divided by possible edges), and similar degree distributions (with mean degree 12.9 for MetaCore™ and 11.6 for iRefWeb, and standard deviations of 26.1 and 21.8 respectively). However, when we considered only the nodes which were common to both networks, the node degrees appeared to be quite uncorrelated, achieving a Pearson correlation of 0.52, which was reduced to 0.095 when we removed the 222 nodes with degree greater than 50.

Using the InfoMap community detection algorithm [3] implemented via the igraph package, we found that the two networks have very different community structures. We identified 406 communities within the MetaCore™ network, with only one community (with 160 members) having more than 100 members. For the iRefWeb network, however, 680 communities were identified, four of which had more than 100 members, with the largest such community having 350 members. The MetaCore™ network with the InfoMap community structure has modularity

equal to 0.42 (modularity is a measure of the quality of the division of the network into communities [4]), while the iRefWeb network has modularity 0.54 with the InfoMap community structure. In practice, it is thought that a modularity above 0.3 is an indicator of significant community structure in a network [4], implying that these community structures identified are accurate. Using various community comparison metrics (including the adjusted rand index [5] and the variation of information metric [6]) we found that the community structures of these networks are extremely dissimilar. In particular, the comparison metric values attained were similar to the values of the metrics attained when comparing the community structures of two randomly generated graphs with the same degree distributions as the MetaCore™ and iRefWeb networks, respectively. Thus, from the information presented above, it seems as though the MetaCore™ and iRefWeb networks are sufficiently different from one another such that the validation of our findings from the iRefWeb network using the MetaCore™ network imply that the network-based methods are reasonably network-invariant.

Comparison of the ovarian cancer data set and the melanoma data set

As described and discussed in the main manuscript, the results obtained from the ovarian cancer data set were somewhat different to the melanoma data set. Following processing and filtering, the melanoma data set consists of 17,552 genes expression probes for 25 GP patients and 22 PP samples. The ovarian cancer data set, on the other hand, consists of 12,981 genes expression probes for 33 GP samples and 39 PP samples. The data sets contain 9,856 genes in common (corresponding to 56% of the genes in the melanoma data set and 76% of the genes in the ovarian data set). Restricting the data sets to the genes appearing in the iRefWeb network reduced the number of genes in the melanoma data set to 5,981 and the number of genes in the ovarian cancer data set to 5,623, approximately 80% of which were common to both. Performing a moderated *t*-statistic DE analysis on each data set, as outlined in the supplementary methods above, we found that the melanoma data set has 96 DE genes (p-value < 0.1) and the ovarian cancer data set has only 13 DE genes.

References

1. Csardi G, Nepusz T: **The igraph software package for complex network research.** *InterJournal* 2006, **Complex Systems:**1695.
2. R_Core_Team: **R: A Language and Environment for Statistical Computing.** Vienna, Austria: R Foundation for Statistical Computing; 2014
3. Rosvall M, Bergstrom CT: **Maps of Information Flow Reveal Community Structure In Complex Networks.** *Proceedings of the National Academy of Sciences* 2008, **105:**6.
4. Clauset A, Newman MEJ, Moore C: **Finding community structure in very large networks.** *Physical Review E* 2004, **70:**066111.
5. Hubert L, Arabie P: **Comparing partitions.** *Journal of Classification* 1985, **2:**193-218.
6. Meilă M: **Comparing Clusterings by the Variation of Information.** In *Learning Theory and Kernel Machines. Volume* 2777. Edited by Schölkopf B, Warmuth MK: Springer Berlin Heidelberg; 2003: 173-187: *Lecture Notes in Computer Science*.
